# DNA methylation dynamics play crucial roles in shaping the distinct transcriptomic profiles for different root-type initiation in rice

**DOI:** 10.1186/s13059-025-03571-0

**Published:** 2025-04-17

**Authors:** Wei Jiang, Zhou Zhou, Xiaoying Li, Yu Zhao, Shaoli Zhou

**Affiliations:** National Key Laboratory of Crop Genetic Improvement, Hubei Hongshan Laboratory, Huazhong Agricultural University, Wuhan, China

**Keywords:** DNA methylation, Crown root, Lateral root, Embryonic root, Initiation, Rice

## Abstract

**Background:**

Monocots possess a fibrous root system comprising an embryonic root, crown roots, and lateral roots. The distinct cellular origins highlight the diversity of the initiation mechanism. To date, the distinct initiation mechanisms have been poorly studied. In this study, we conduct a comprehensive transcriptome and DNA methylome assay of these root types during their initiation.

**Results:**

Our findings indicate significant divergence in transcriptome regulation trajectories with apparent transcriptional activation in post-embryonic root initials (crown root and lateral root) contrasted by suppression in embryonic root generation. Additionally, CHH methylation is dynamically and differentially regulated across the initiation stages of the various root types, and is significantly associated with the short transposon element within the promoter regions of functional genes, which plays crucial roles in determining the genes’ spatiotemporal transcription. Moreover, our work reveals that the activation of *DNA glycosylase 702 (DNG702)* and repression of *Domains Rearranged Methyltransferase 2 (DRM2)* play important roles in the erasure of CHH methylation and activation of functional genes during the processes, such as a novel identified key regulatory *bZip65*, thus directly impacting the initiation of post-embryonic roots in rice.

**Conclusions:**

Our extensive analysis delineates the landscapes of spatiotemporal transcriptomes and DNA methylomes during the initiation of the three root types in rice, shedding light on the pivotal role of CHH methylation in the spatiotemporal regulation of various key genes, ensuring the successful initiation of distinct root types in rice.

**Supplementary Information:**

The online version contains supplementary material available at 10.1186/s13059-025-03571-0.

## Background

Plant roots are essential vegetative organs that anchor plant and absorb water and nutrients from the soil. Cereals, like rice (*Oryza sativa*), maize (*Zea mays*), and wheat (*Triticum aestivum*) possess a fibrous root system composed of a primary root (PR), postembryonic crown roots (CRs) and lateral roots (LRs). The PR is ephemeral and short-lived, developing from the embryonic root (ER) after seed germination. CRs, derived from pericycle-like cells in the stem or coleoptile nodes, are the main components of the monocot root system. They are continuously replenished and reshaped throughout the plant’s life cycle [1, 2]. While LRs, which originate from the pericycle cells of CR or PR, contribute to the root system volume [3].

Over the past years, several key regulatory factors for rice root, such as the key genes for crown root, *CROWN ROOTLESS1* (*CRL1*), *WUSCHEL* (*WUS*)-*RELATED HOMEOBOX11* (*WOX11*), *Cytokinin-Responsive Regulator2* (*OsRR2*), *SQUAMOSA PROMOTER-BINDING PROTEIN-LIKE3* (*SPL3*), and *YUCCA-like gene 1* (*YUCCA1*), and the key genes for lateral root, *LATERAL ROOTLESS2* (*LRT2*), *CASEIN KINASEI1*(*CKI1*), *auxin influx carrier 1* (*AUX1*), and *origin recognition complex protein 3* (*ORC3*), have been identified and characterized to function in different stages of rice postembryonic root formation [2, 4–11]. Most of these factors are essential for either CR or LR formation, combining with the divergent origins of the three root types, implying the different initiation mechanisms of the three type roots. To date, it still lacks a comprehensive study of the transcriptome dynamics during the initiation of different root types in cereals, limiting our understanding of different root initiation mechanisms.

Cytosine methylation, a key epigenetic modification, occurs mainly in three distinct cytosine contexts in plants: CG, CHG, and CHH (where H is A, C, or T). This modification is stably inherited across generations and is dynamically regulated by DNA methylases (such as DOMAINS REARRANGED METHYLTRANSFERASE 2, DRM2) and DNA glycosylases (such as DNG family members) which play pivotal roles in cytosine methylation erasure [12–15]. Loss-of-function of *DRM2* led to a ~ 85% decrease in CHH methylation in rice somatic tissue, causing misregulated genes and severe developmental phenotypes [16].Conversely, mutants of DNG family members (e.g., DNG702 and DNG701/4) showed significant elevation in DNA methylation across the rice genome, impacting gene transcription and organ development, such as embryogenesis, endosperm, and pollen development [14, 15, 17, 18].

DNA methylation is dynamically regulated during organogenesis. In *Arabidopsis*, analysis of DNA methylation in six distinct cell types of root apical meristem (RAM) revealed CHH hypermethylation of transposon elements (TEs) in the columella, potentially associated with columella functions [19]. CHH methylation significantly increased throughout the seed development in soybean and *Arabidopsis*, primarily targeting transposons [20]. The development of the gynophore was associated with DNA methylation dynamics contributing to the light response and auxin biosynthesis [21]. However, to date, DNA methylation patterns during the root initiation process and whether and how DNA methylation is involved in gene expression regulation during root initiation remain unknown in rice. Understanding these dynamic regulatory processes will help establish a better framework for modeling crop root formation.

Here, we present a comprehensive analysis of genome-wide transcriptomes and DNA methylomes at representative stages during the initiation of CR, LR, and ER in rice. Our findings reveal that the three root types possess distinct transcriptional regulation trajectories (or initiation mechanisms) and key regulators during the initiation phase, suggesting diverse initiation mechanisms. The genomic activation of CR and LR and repression in ER are associated with global CHH methylation changes. Additionally, the dynamically regulated DRM2 and DNG702 directly impact key gene transcriptions and eventually determine root primordia generation via CHH methylation regulation of the short transposons within the promoters. Our work enhances the understanding of rice root initiation mechanisms and provides a valuable resource for future functional analyses of rice root system formation.

## Results

### Spatiotemporal transcriptome profiling reveals distinct regulation mechanisms underlying the initiation of ER, CR, and LR

**Fig. 1:**
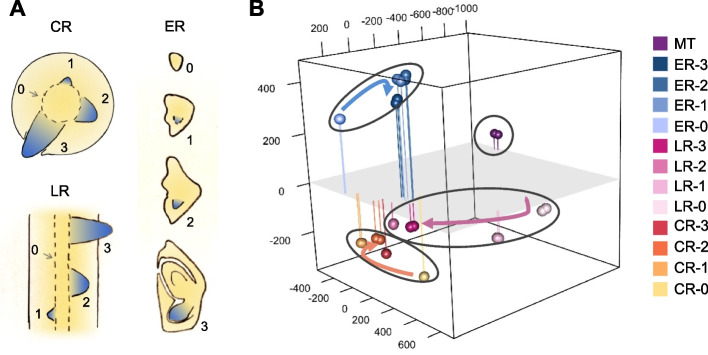
Spatiotemporal transcriptome profiling of different root initiation stages in rice. **A** Schematic diagrams showing different stages of root initials profiled in this study. Stage 0 for crown (CR), lateral (LR), and embryonic (ER) roots indicates the pericycle-like, pericycle, and globular embryo cells, respectively. **B** Multidimensional scaling analysis of transcription profiles of different root initial samples during morphogenesis. MT, mature root tips. Vertical lines illustrate the distances between points and the plane (*z* = 0)

In rice, ER, CR, and LR exhibit anatomical resemblances at the maturity (for CR and LR) or adult stage (for ER after germination). However, the distinct origination from different cellular backgrounds suggests the diverse initiation mechanisms for the three root types (Fig. 1A and Additional file 1: Fig. S1). To gain a comprehensive insight into the initiation mechanisms of different rice roots, we conducted stage-specific transcriptome analyses on the developing root initials by laser microdissection. The developmental stages were categorized into four stages (S0, S1, S2, and S3) based on the established literature [22, 23]. Among these, S0 represents the undifferentiated cells (pericycle or pericycle-like cells for LR and CR, globular embryo for ER), while S1 encompasses the newly developed initial cell clusters without obvious cell divergence, S2 corresponds to the initials prior to emergence, when the initials have achieved cell layering comprising stele, cortex, epidermis and root cap, and S3 represents the emerging initials with further developed and formed cell layers, respectively (Additional file 1: Fig. S1B to D and S2).

Two independent biological replicates were performed for each sample. Data quality evaluation analysis shows the high repeatability of the biological replicates, with an average of 33.5 million unique mapped reads per sample (Additional file 2: Table S1). To further uncover the mechanisms The reliability of our data was further supported by the expression patterns of well-documented genes related to CR or LR development, such as *CRL1*, Os*RR2*, *WOX11*,* SPL3*, and *LRT2* (Additional file 1: Fig. S3) [1, 4–6, 8]. *CRL1*, a key regulator gene for rice crown root [4], was found to be specifically upregulated in CR initials but not in LR and ER. *OsRR2*, a known factor for crown root development [1], was highly expressed at the S0 of CR but silent at later stages, consistent with reported transcription patterns. As the suppressor of *OsRR2*, *WOX11* was found activated after crown root emerging at stage 3 compared to stage 1 and 2 [1]. *LRT2*, a key gene for lateral root formation, is known to be highly expressed in young lateral root primordia but suppressed before root emergence [8]. In our data, it was found to be activated at S1 but repressed at S0, S2 and S3. These results confirmed the dependability of the obtained spatiotemporal transcriptomes for extensive further analysis.

Multidimensional scaling (MDS) was applied to visualize the transcriptome datasets within a three-dimensional space, which illustrated the developmental trajectories and their relationships [24–26]. As shown in the plot, all paired replicates are tightly clustered together confirming the RNA-seq’s repeatability. Intriguingly, the transcriptome clusters of CR and LR at stage 0 (S0) and stage 1 (S1) showed considerable divergence, but gradually approached each other at the later stages (S2 and S3) (Fig. 1B), suggesting their different initial mechanisms and unique expression profiles at the early stage but a convergence of gene expression after root structure formation. Contrary to CR and LR, the transcriptomes of ER during all the stages were consistently distal from those of CR and LR (Fig. 1B), emphasizing the uniqueness of embryonic root initiation. Notably, the transcriptomes of the mature root tips [2, 27] were distinctly separated from all of the transcriptomes of the three root types (Fig. 1B), implying a further extensive transcription remodeling during root elongation and maturation.

### Divergent genes involved in the initiation of ER, CR, and LR

**Fig. 2:**
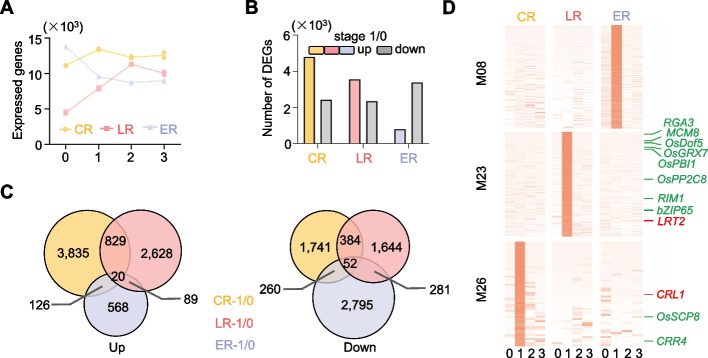
Divergent gene activation during the initiation of three root types.** A** Number of transcribed genes identified at indicated stages of root initials. **B** Summary of differentially expressed gene numbers during root initiation. Stage 1/0: stage 1 versus stage 0. Upregulated genes are represented in three different colors, with downregulated genes in gray. **C** Venn diagrams showing overlaps of up- (left) and downregulated (right) genes in the first stages of the three root types. **D** Heat maps showing the expression profiles of three indicated modules identified by WGCNA. Red indicates reported functional genes, and green indicates others

By comparing the transcribed gene numbers (RPKM > 1), we observed an increase in the number of the expressed genes at the first stage of both CR and LR, with a further elevation in expressed gene number at stage 2 for LR (Fig. 2A). On the contrary, there was a significant reduction in the number of transcribed genes during the development of ER (Fig. 2A), which likely reflects cell specialization during the formation of radical tissue from globular embryo. These findings suggested that the initiation of CR and LR both involved genome activation, whereas ER formation was associated with genome suppression. Consistent with the above observations, 4810 and 3566 genes are identified as upregulated during CR and LR initiation, whereas only 2437 and 2361 genes are downregulated during this process, respectively (Fig. 2B and Additional file 1: Fig. S4 A and B). However, the downregulated genes in ER initials, in comparison to its mother tissue, far exceed the numbers of upregulated genes (3388 versus 803) (Fig. 2B and Additional file 1: Fig. S4B).

Although genome activation was observed during the initiation of both CR and LR, the sets of genes that were up- or downregulated in CR and LR rarely overlapped (Fig. 2C and Additional file 1: Fig. S4 C and D), indicating distinct molecular regulation mechanisms during different root initiations. To identify the potential functional genes for different root types, weighted correlation network analysis (Weighted Gene Co-Expression Network Analysis, WGCNA) was applied to find clusters of highly correlated genes (see the “Methods” section). In summary, 29 modules were identified within the differentially expressed genes (Additional file 1: Fig. S5), among which, 53 (module 26), 504 (module 23), and 322 (module 08) genes were found to be expressed only at the first stage of ER, CR, and LR initials, respectively (Fig. 2D), indicating the divergence of key genes for different root initiation. Among these genes, *CRL1* and *LRT2* were identified as stage 1 specifically expressed genes for CR and LR (Fig. 2D), respectively. Additionally, many other genes, for instance, transcription factor genes like *bZIP65*,* OsDof5*, and *RIM1* were clustered within these modules, implying their potential roles in root initiation regulation (Fig. 2D).

### Global CHH methylation changes during rice root initiation

DNA methylation plays a critical role in regulating gene transcription [28, 29], as well as in the differentiation and development of plant organs [17, 30]. To investigate the relationship between transcription dynamics and DNA methylation during rice root initiation, we conducted low-input Bisulfite-sequencing (BS-seq) for the three developmental stages (S0, S1, and S2) for each type of root. Two replicates were performed for each stage, with 17–34 million uniquely mapped reads generated for each sample, representing 25–50 times coverage of the genome (Additional file 3: Table S2). The correlation coefficients of the replicates for global levels of DNA methylation were greater than 0.9 (Additional file 3: Table S2), suggesting the high quality of our data.

**Fig. 3:**
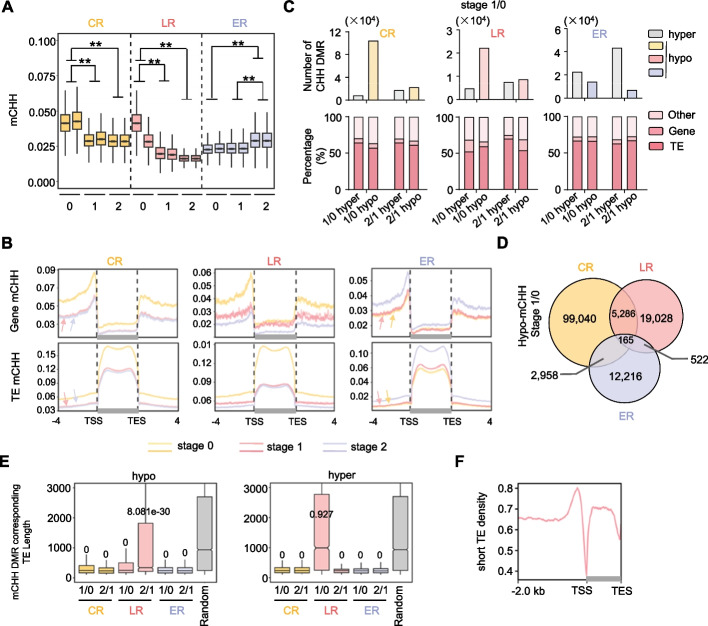
Global CHH methylation is diversely regulated during the initiation of three root types.** A** Box plots indicate the methylation ratios at the CHH context across the genome. Methylation of each 100 kb was summarized. Two biological replicates are shown for each sample. The 25 th and 75 th percentiles (box), median and highest and lowest values are shown. Double asterisks indicate significant differences, *P*-value < 0.01 (Student’s *t*-test). **B** Metaplots showing the average CHH methylation levels on genes, transposon elements, and their flanking regions. **C** Upper panel, histograms showing numbers of differentially CHH methylated regions (DMR) identified in indicated comparisons. Lower panel, genomic distribution of DMRs. TE, transposon elements. **D** Venn diagram shows the overlap of hypo-CHH DMRs identified in the initials of three root types. **E** Box plots summarizing the length distribution of CHH DMRs associated transposon elements. Random selected genomic 50 bp windows were used as control. **F** Metaplot showing enrichment of short transposon elements (< 500 bp) across the genic regions in the rice genome

Analysis of global levels of DNA methylation showed that mCG and mCHG are relatively stable across the different developmental stages for all three root types (Additional file 1: Fig. S6 A and B). In contrast, mCHH underwent massive changes during root initiation (Fig. 3A). Specifically, in CR, the global mCHH level at stage 1 was significantly decreased by 34.06% ((5.02–3.31%)/5.02%, *z*-test, *P* value = 0) compared to that at S0 (Fig. 3A), with no significant difference between S2 and S1. For LR, the CHH methylation was decreased by two sequential steps, 44.81% ((3.37–1.86%)/3.37%) for S1/S0 and 19% ((2.11–1.71%)/2.11%) for S2/S1 (*z*-test, *P* value = 0) (Fig. 3A). Conversely, mCHH was found to increase by 28.28% ((3.13–2.44%)/2.44%) at S2 compared to that at S0 in ER (*z*-test, *P* value = 0) (Fig. 3A).

Investigation into methylation across genes and transposon elements revealed a predominance of CHH methylation decreased at the flanking regions of genes relative to the body regions (Fig. 3B). On the contrary, CHH methylation tends to change at the body regions of the transposon element rather than their flanking regions (Fig. 3B). Scans of differentially methylated regions (DMR) within 50 bp windows across the genome (see the “Methods” section) revealed much more dynamic methylation changes in CHH context compared to CG and CHG during the initial stages of root development (Fig. 3C, Additional file 1: Fig. S6 C and D, Additional file 4: Table S3), consistent with the global methylation trends (Fig. 3A, Additional file 1: Fig. S6 A and B). Further analysis revealed that significantly more hypo DMRs (104,430 and 22,208) were identified relative to hyper DMRs (8,401 and 4,811) during the initiation stage (S1/S0) in both CR and LR, but the opposite pattern observed in ER (Fig. 3C, Additional file 4: Table S3). Moreover, the hypo DMRs identified in the ER, CR, and LR were rarely overlapped (Fig. 3D, Additional file 1: Fig. S7 A), suggesting the distinct DNA methylation regulation manner during different root initiation, consistent with the observation of rarely overlapped DEGs (Fig. 2C). Intriguingly, except for the hyper DMRs of LR 1/0, CHH DMRs were found significantly associated with short TEs less than 500 bp in length compared the control of randomly distributed windows (Fig. 3E, Additional file 1: Fig. S7B) (see the “Methods” section). These short TEs are enriched at the promoter regions of functional genes and reported to be associated with gene transcription regulation (Fig. 3F) [31, 32], suggesting the potential function in gene transcription regulation by CHH methylation dynamics during root initiation.

### Divergent CHH methylation regulation is involved in the gene transcription dynamics in CR and LR initials

**Fig. 4:**
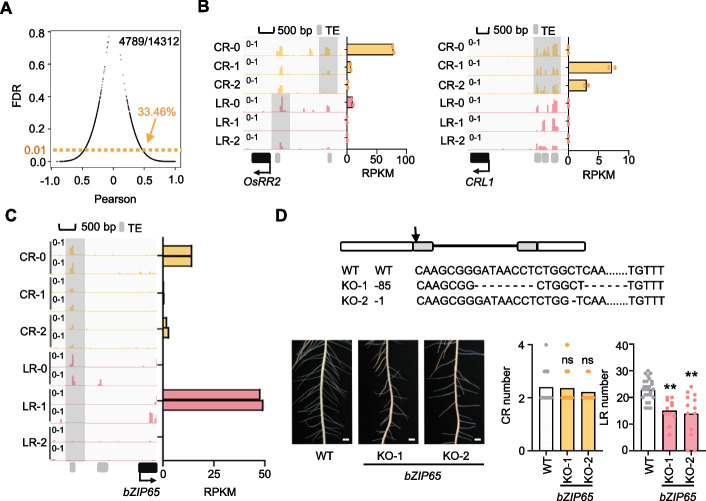
CHH methylation shapes key gene transcription for root initiation. **A** Analysis of correlations between gene transcription and CHH methylation of TE within the corresponding promoters. 14,312 genes were identified with transcription variation (see the “Methods” section) during root initiation, while 33.46% (4,789/14,312) were significantly correlated with CHH methylation dynamics. **B** and** C** IGV screenshots showing the CHH methylation dynamics within the promoter regions of indicated genes during crown and lateral root initiation. Histograms show corresponding transcription levels. Two biological replications are represented. **D** Knock-out of *bZIP65* decreases lateral root numbers in rice without affecting crown roots. Upper panel, schematic diagrams show the CRISPR target (arrow) and the mutants generated by using CRISPR-Cas9 system. Two frameshift homozygous lines are shown. Lower panel, statistics of crown and lateral root numbers compared to wild type (WT). Double asterisks indicate significant differences, *P*-value < 0.01 (Student’s *t*-test). ns, no significant difference. *N* = 15, 22, and 10 for CR, 27, 11, and 13 for LR

To survey the effect of DNA methylation on gene expression, we investigated and found more than half of the DEGs during the root initiation are associated with CHH methylated promoter (Additional file 1: Fig. S8 A) (see the “Methods” section). Among the genes with transcription variation during the root initiation, approximately 33.46% of the gene transcription was found to positively or negatively correlate with CHH methylation of the TE within the corresponding promoter regions (Fig. 4A, Additional file 5: Table S4) (see the “Methods” section). This suggests that DNA methylation influenced a part of genomic gene transcription in both directions as reported [28, 33]. Nearly 70% of genomic genes showed no significant correlation between DNA methylation and transcription, implying that other factors, in addition to DNA methylation, were also involved in the regulation during these processes.

A closer inspection of the correlated genes unveiled a series of reported genes involved in root development that were regulated by CHH methylation (Additional file 5: Table S4). For instance, *OsRR2*, which is reported as a key gene expressed at the early stage of crown root [1], showed transcription positively associated with CHH methylation of a short TE within its promoter region (Fig. 4B). Similarly, *CRL1* and *WOX11*, the key gene for rice crown root development [1, 4], were found to be regulated by CHH methylation within its promoter regions which negatively impacts gene transcription (Fig. 4B, Additional file 1: Fig. S8B). In addition to the reported genes, several genes essential for the fundamental cellular activities (e.g., *Tubulin* and *Ribosome Large Unite 9*), were also found to be associated with the CHH methylation regulation in LR, but not in CR (Additional file 1: Fig. S8B), suggesting the specific DNA methylation regulation pattern in different root primordia. For LR, we investigated the genes exhibiting specific transcription patterns during root initiation (Fig. 2D). Several transcription factors (e.g., *bZIP65*,* OsDof5*,* OsBBX22*, and *OsARF7*) were found to exhibit a strong negative correlation between the CHH methylation of TEs within their promoter regions and gene transcription (Fig. 4C and Additional file 1: Fig. S8 C). For instance, the CHH methylation at the *bZIP65* promoter was specifically removed during LR initiation but not in CR, with its transcription being temporally activated at stage 1 (S1) of LR, highlighting its potential role in promoting lateral root development (Fig. 4C). To validate this, we generated knock-out lines and discovered that the loss-of-function mutant of *bZIP65* indeed affects lateral root formation, without significantly effecting crown roots (Fig. 4D). Notably, CHH methylation was absent in the later stage (LR- 2) as well, however, *bZIP65* was not expressed at LR- 2, suggesting complexity in gene transcription regulation within the cells. In summary, our findings demonstrate that spatiotemporal-specific regulation of CHH methylation at short TE sites within the promoters of indicated key genes likely influences rice root initiation by modulating their transcription.

### *DNG702* and *DRM2* involve in rice crown and lateral root development

**Fig. 5:**
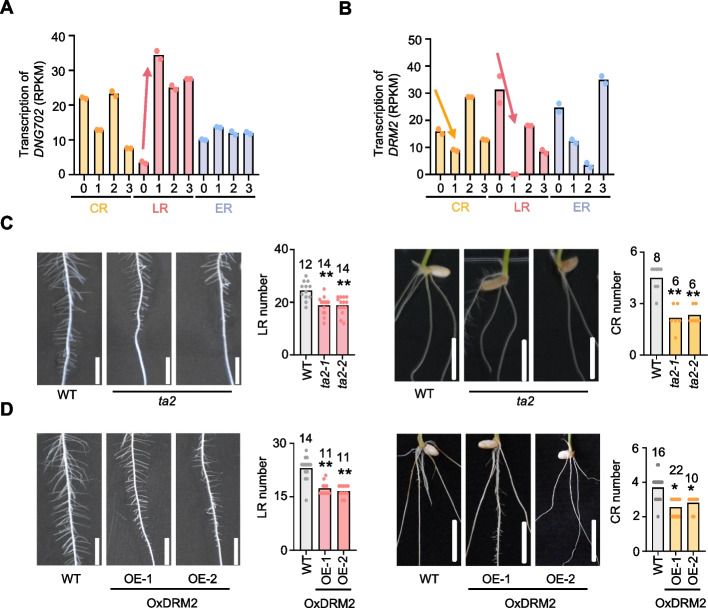
DNA demethylase DNG702 and methylase DRM2 role in CR and LR development.** A** and** B** Histograms showing the transcription dynamics of *DNG702* (**A**) and *DRM2* (**B**) during crown, lateral, and embryonic root initiation. Two biological replicates are indicated. Arrows indicate the transcription trend during initiation. **C** and** D** Point mutant of *DNG702* (*ta2*) (**C**) and overexpression of *DRM2* (**D**) both affect rice crown and lateral root initiation. Histograms show the statistics of crown and lateral root numbers. Two biological replicates for *ta2* mutant and two independent transgenic lines for *DRM2* overexpression are performed. Tested numbers for each sample are indicated. Double and single asterisks indicate significant differences, *P*-value < 0.01 and < 0.05 (Student’s *t*-test). ns, no significant difference. Bars = 0.5 cm and 1 cm for LR and CR, respectively

The activation of the DNA demethylation pathway or the attenuation of the methylation pathway both lead to the reduction of global DNA methylation. To uncover the mechanisms underlying CHH methylation loss during post-embryonic root initiation, we investigated the transcription profiles of genes associated with DNA methylation (Additional file 1: Fig. S9 A and B). We identified that *DNG702* was significantly upregulated during the initiation of LR, while *DRM2* was decreased in both the stage 1 (S1) of CR and LR initials (Fig. 5A and B), suggesting the potential roles of *DNG702* activation and *DRM2* repression in DNA methylation reduction in CR and LR.

Due to the lethality of the *DNG702* knockout mutant [15], we inspected the root phenotypes using a point mutant (*ta2*) that leads to alternative splicing for *DNG702* [34], as well as the overexpression lines of *DRM2* (Fig. 5D and Additional file 1: Fig. S9 C). According to the statistics, the *ta2* mutant and *DRM2* overexpression, which theoretically increase global DNA methylation, showed a significant decrease in the numbers of both CR and LR and their corresponding initials (Fig. 5C and D, Additional file 1: Fig. S10). This suggests the important role of DNA methylation reduction in crown and lateral root initiation. Furthermore, we investigated the impact of the *drm2* mutant which largely loses DNA methylation in its genome. The results revealed significant developmental deficiencies in both crown and lateral root (Additional file 1: Fig. S9D), likely due to the indirect effects of globally misregulated genes and severe developmental phenotypes reported for the *drm2* mutant [16].

### DNG702 and DRM2 coordinately regulate key genes activation in CR and LR initials

**Fig. 6:**
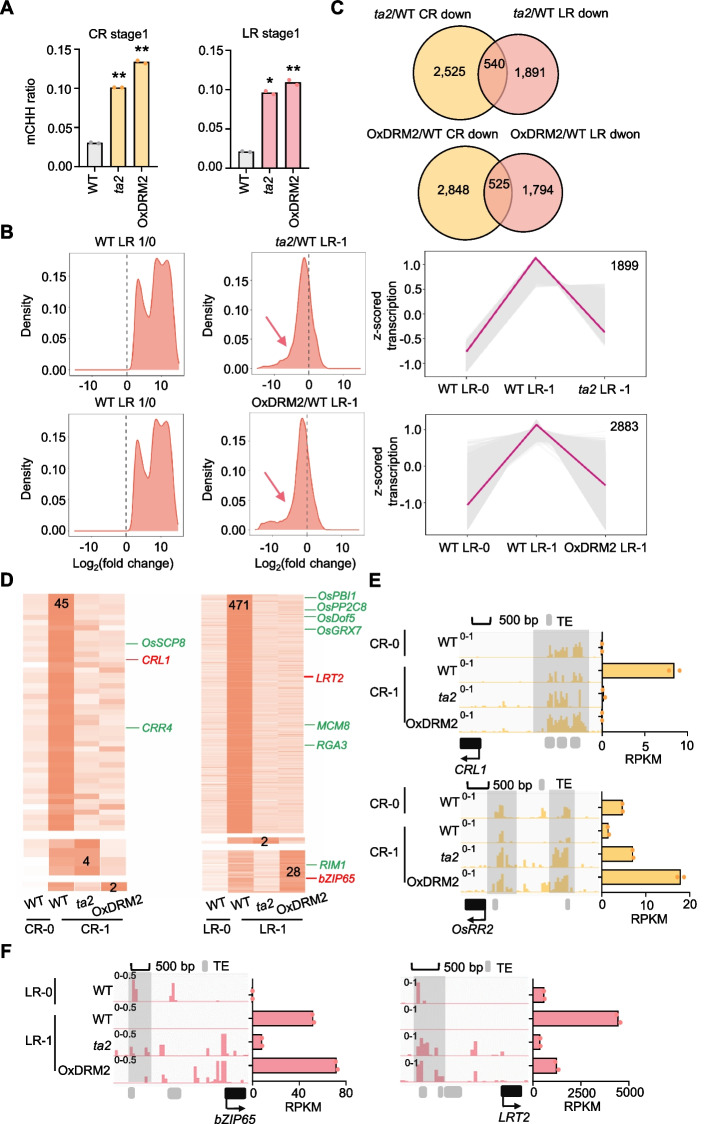
DNG702 and DRM2 are involved in transcriptional regulation of key genes essential for CR or LR initiation.** A** Global CHH methylation increase in *ta2* mutant (*DNG702* point mutant) and *DRM2* overexpression lines compared to wild type. Double asterisks represent significant differences, *P*-value < 0.01 (Student’s *t*-test). **B** Parts of the activated genes in LR- 1 are not successfully activated in *ta2* mutant and *DRM2* overexpression lines. Left panel, density plots of fold change for LR- 1/0 upregulated genes. Middle panel, density plots of fold change for the indicated mutant versus wild type at stage 1. Right panel, gene clusters of which transcription was not successfully activated in *ta2* mutant (upper panel) or *DRM2* overexpression lines (lower panel). Gray lines represent each gene, red line indicates fitted curve. Gene numbers are shown. **C** Venn diagrams show the overlaps of downregulated genes in CR and LR for *DRM2* overexpression lines (upper panel) and *ta2* mutant (bottom panel). **D** Clustered heat maps showing transcription profiles of genes specifically expressed at stage 1 of CR (left panel) and LR (right panel). **E** and** F** IGV screenshots show the CHH methylation dynamics in wild type and the indicated materials. Histograms show the corresponding gene transcription

To further uncover the mechanisms by which the *ta2* mutant and *DRM2* overexpression affect CR and LR development, we performed transcriptome and DNA methylome assays for stage 1 using laser-microdissection coupled with high-throughput sequencing as the wild type mentioned above. As expected, DNA methylation, particularly CHH methylation significantly increased in both materials compared to the wild type (Fig. 6A, Additional file 1: Fig. S11 A and B). By inspecting gene transcription, we found many genes (1899 and 2883 for LR, 1226 and 1649 for CR), which should be activated at stage 1 of CR or LR, were downregulated in the *ta2* mutant and *DRM2* overexpression lines (Fig. 6B and Additional file 1: Fig. S11 C). This suggested the role of DNG702 and DRM2 in shaping the transcriptome at the initial stage. Gene ontology analysis showed these misregulated genes were enriched in several regulatory ontologies involved in gene transcription regulation, RNA processing, protein translation, etc., indicating the fundamental cellular processes needed by the proliferation cells are not successfully activated in the *ta2* mutant and *DRM2* overexpression lines(Additional file 1: Fig. S11D and E). Further investigation into the misregulated genes, revealed that the down- or upregulated genes rarely overlapped in LR and CR (Fig. 6C and Additional file 1: Fig. S12 A), indicating that DNG702 (or DRM2) regulates divergent genes in CR and LR. Moreover, to our surprise, we found that the majority of the genes specifically expressed in CR- 1 or LR- 1 failed to activate in the *ta2* mutant and *DRM2* overexpression lines (Figs. 2D and 6D), highlighting the role of DNG702 and DRM2 in activating the genes essential for the CR and LR initiation. Notably, some key known regulators for CR and LR (e.g., *CRL1*, *OsRR2*,* LRT2*) were identified within the misregulated genes (Fig. 6E, F, and Additional file 1: Fig. S12B), suggesting that DNG702 and DRM2 potentially determine CR and LR initiation by regulating transcription of key genes through DNA methylation manipulation. Additionally, *bZIP65*, a key gene for rice lateral root identified in our work (Fig. 4C and D), was found to be only transcriptionally regulated by DNG702 rather than DRM2 (Fig. 6F), implying these two regulators are distinct in regulating part of genes in the genome. To determine whether the transcriptional regulation of the indicated genes was directly mediated by DNG702, we collected the coleoptile nodes and roots comprising initiating CR and LR primordia and performed the Cut&Tag-qPCR sequentially. As shown in Fig. S12 C, DNG702 binding signal was significantly enriched at the loci of target genes in the tissues with CR and LR primordia, relative to the reference gene (*GAPDH*) and wild type control. These results implied that DNG702 directly affected the transcription of indicated key genes.

## Discussion

Cereals, as monocotyledons, possess a fibrous root system that is crucial for the uptake of water and nutrients, ultimately influencing crop yields. To date, amount of functional genes have been identified to function in either crown or lateral root formation [2, 4, 5, 8]. Moreover, studies leveraging single-cell or spatial transcriptomics have provided insights into the gene expression patterns across various root cell types [19, 35, 36]. However, there has been a lack of systematic analysis to elucidate the regulatory networks involved in root initiation. The distinct origins of embryonic (ER), crown (CR), and lateral (LR) roots from different cells or tissues suggest unique initiation mechanisms. To this end, we conducted a spatiotemporal analysis of transcriptomes and DNA methylomes during the initiation phases of all three root types.

Our transcriptome trajectory assay revealed distinct clustering between the embryonic and post-embryonic roots, as well as between crown and lateral root initials. This indicated diverse regulatory mechanisms governing the generation of different root primordia. Using WGCNA analysis, we identified a series of spatiotemporal- specifically expressed genes (Fig. 2D). Apart from *bZIP65* which has been proven to be involved in LR formation in our work (Fig. 4C and D), a series of functional genes specifically expressed during rice root initiation were investigated as well to validate their potential roles in rice root formation. Among them, *CBP1* (*calmodulin-binding protein 1*), with predominance expression at stage 1 and 2 of CR initials, was found to be instrumental in crown root initiation, and had no significant effect on lateral roots (Additional file 1: Fig. S13 A to C). However, *ERF69*, *PIN9,* and *DREB2B* showed no obvious effects on either crown or lateral roots, despite their relatively specific transcriptional patterns. Our findings thus contribute valuable datasets for identifying key regulators of rice root initiation.

In our study, we noticed an increase in the number of transcribed genes in CR and LR initials, with a decrease during ER formation (Fig. 2A). Upon the formation of CR or LR, pericycle or pericycle-like cells undergo dedifferentiation to generate primordia for post-embryonic roots. We hypothesize that the transition of cell fate from mature tissue to meristems with rapid cell division necessitates the activation of a set of genes, including those involved in nucleic acid and protein metabolism (Additional file 1: Fig. S11D and E). Unlike CR and LR formation, ER emerges from the globular embryo, which possesses high totipotency, and sequentially dehydrates during seed maturation. This suggests that the ER genome might be gradually suppressed during this process.

Epigenetic mechanisms are reported to be implicated in the regulation of genomic status and gene transcription. In our investigation, we explored the DNA methylation dynamics during various root initiation processes. Surprisingly, we found a perfect negative correlation between global CHH methylation levels and the genome activation status (Figs. 2A and 3A), despite DNA methylation being known to bidirectionally influence gene expression. Specifically, the reduction in CHH methylation at stages 1 and/or 2 was accompanied by an increase in the number of expressed genes in CR and LR initial, and on the contrary, the increase in CHH methylation during ER formation correlated with a reduction in the number of expressed genes. Thus, we propose that the decrease in global CHH methylation levels is necessary for the meristem cells engaged in active biological processes, a finding echoed in other studies [37, 38]. There was no globally consistent relationship between CHH methylation and transcription during organogenesis (Additional file 1: Fig. S14), with only 33.46% showing significant correlation. This suggested the complexity of transcriptional regulation mechanisms during these processes.

Contrary to the patterns observed in post-embryonic root development, DNA methylation increased during ER formation (Fig. 3A). Inspection of genes related to DNA methylation, *AGO4,* and *RDR2*, key members involved in RdDM pathway, were found activated during ER genesis (Additional file 1: Fig. S9E). This potentially explains the increase of DNA methylation in ER. Additionally, a subset of genes specifically expressed in ER were found to be influenced by CHH methylation dynamics (Additional file 5: Table S4), emphasizing the pivotal role of DNA methylation dynamics in establishing spatiotemporal transcription patterns during root initiation. For instance, *waf1* exhibited a malformed radicle during embryogenesis [39], and *Os02 g28850*, a homolog of *NtKRP*, which impacts the embryonic root length in tobacco [40], were both found to be up-regulated and CHH methylation-regulated during ER organogenesis (Additional file 1: Fig. S9 F). The role of DNA methylation in shaping the formation of embryonic roots requires further investigation.

DRM2 is a key regulator in the RdDM pathway associated with small RNAs. Apart from DNA methyltransferase DRM2, we observed modest transcriptional changes in genes involved in small RNA generation and recognition. For instance, *RDR2* showed decreased expression in CR initials and *AGO4a* exhibited decrease expression in LR initials (Additional file 1: Fig. S9E). These findings suggest potential alterations in small RNA generation pathway during root initiation, influencing DNA dynamics in these processes.

Despite no upregulation trend observed in CR initials for *DNG702* transcription compared to LR, the *ta*2 mutant showed significant impacts on lateral root development (Figs. 5A and 6F). We hypothesize that DNG702 deposition to genomic regions were largely shuffled during LR initiation, a hypothesis that requires further evidence in future research.

In addition to the direct impact of CHH methylation, as shown in Fig. S12 C, the misregulated genes in *ta2* and *OxDRM2* could possibly be caused by changes in higher-layer genes.

While CHH methylation dynamics were present in the developmental processes of all three types of rice roots, they rarely overlapped (Fig. 3D and Additional file 1: Fig. S7 A). For instance, CHH methylation was specifically removed at the stage 1 of LR at the loci of *bZIP65, OsDof5,* and *OsBBX22*, with no significant change in CR. When combined with the overlapping patterns of differentially expressed genes (Fig. 2C, Additional file 1: Fig. S4 C and D), these findings underscore distinctive chromatin modification and gene expression regulation patterns in the three rice root types, and imply discrete initiation mechanisms among them. The evidence from *ta2* mutant and *DRM2* overexpression lines further supports this hypothesis. Despite DNG702 and DRM2 appearing to affect a same set of genes (Fig. 6D), the genes they regulated in CR- 0 and LR- 1 are largely distinct (Fig. 6C and Additional file 1: Fig. S12 A), suggesting they regulate different gene sets in CR and LR initials.

Noticeably, LRs are classified into small (S-LR) and large (L-LR) types in rice, S-LRs are relatively short and thin, compared with the long and thick L-LR, which often produce higher-order LRs (Additional file 1: Fig. S15 A and B) [41]. Within our study, we focused solely on the initiation of S-LR. Further studies are required to determine whether L-LR has distinct transcriptome trajectories and CHH methylome regulation patterns.

## Conclusions

**Fig. 7:**
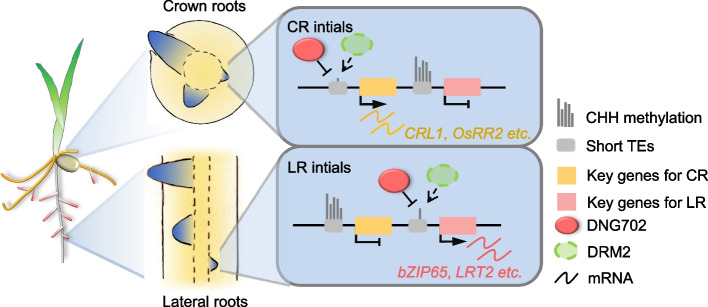
Model illustrating DNG702 and DRM2 involvement rice crown and lateral root initiation. During crown and lateral root initiation, downregulated *DRM2* and upregulated *DNG702* lead to decreased DNA methylation at key regulatory genes, activating key genes’ transcription and accelerating root initiation. DNG702 and DRM2 share similar target genes in CR and LR, however, the downstream genes in CR and LR are distinct

In summary, our investigation aimed to conduct a comprehensive analysis of the mechanisms underlying the initiation of three different root types in rice. We found that cellular reprogramming, involving gene transcription and DNA methylation, underwent uniquely and distinctly for each root type. CHH methylation dynamics, particularly affecting short transposon elements within promoter regions, played a crucial role in shaping spatiotemporal transcription patterns for key genes involved in the generation of root initials. Additionally, the transcription dynamics of *DNG702* and *DRM2* merged as significant regulators of DNA methylation during these biological processes, influencing distinct downstream genes in CR and LR (Fig. 7). The data presented in our work enhance the understanding of the formation mechanisms of different root primordia, providing further insights into the nature of root initiation. Furthermore, the comprehensive datasets on transcription and DNA methylation presented in this study will serve as valuable resources for the scientific community, fostering further exploration into the complexities of rice root formation.

## Methods

### Plant materials and growth conditions

Rice (*Oryza sativa* spp *japonica/geng*) material used in this study is from “Zhonghua11” (ZH11) background. For germination culture, seeds were surface-sterilized and germinated in medium containing 0.8% agar supplemented with 3% (w/v) sucrose at 28 °C (in light) and 24 °C (in dark) with a 14-h-light/10-h-dark cycle. For growth in field, germinated rice seedlings were transferred to greenhouse at 25–30 °C under 14-h-light followed by 10-h-dark. The overexpression transgenic plants were produced using the ubiquitin promoter vector pU1301 - 3 × FLAG [42, 43]. The knock out lines (T1 generation, Cas9-free) generated by CRISPR/Cas9 system. The point mutant of *DNG702* was from the reported work [34].

### Microdissection

The specific stages of different primordia were referred to the classification as reported previously [22]. To benchmark the developmental stages for the three root-type initials, S1 refers to the initials containing cell clusters without obvious cell differentiation. In the contrast, S2 contains more noticeable differentiated root layers comprising stele, cortex, epidermis and root cap. For S3, the roots were further developed resulting in more cell layers and fine structures (Additional file 1: Fig. S2). In terms of primordium size, the lengths of S1, S2, and S3 for the crown root initials are 80, 200, and 500 µm, and those for the lateral and embryonic roots are 40, 80, and 200 µm, respectively. LR initials were collected from the roots 7–10 days after germination in the medium. Under the watery condition and during the early development period, the majority of LR initials are of the S-LR type (Additional file 1: Fig. S15 A and B). Only S-type LR initials were collected in this work [41].

The method for preparing paraffin-embedded sections of the primordia of crown, lateral, and embryonic root generally followed the steps described by Takahashi et al. (2010) [44] with minor modifications. The samples with CR and LR initials were collected from the roots and internodes of coleoptile, 4–5 and 7–10 days after germination, respectively. All samples were collected at 9 a.m. Canonical paraffin-embedding is a time-consuming process that generally lasts for one week. In this work, to avoid the degradation of RNA and genomic DNA in the minor tissues, a rapid embedding procedure was conducted, which could be achieved within one day. In general, all the following solution infiltration steps were carried out via an oven-heating water bath to speed up the biochemical exchange between the tissue and solution (Additional file 1: Fig. S15D), the real-time working power of the microwave oven was set at 400 W. The samples were microwaved and fixed in pre-cooled Farmer’s fixative solution (ethanol:acetic acid 3:1) for three times, with the temperature kept around 37 °C (15 min for each step, with fresh fixative solution used for each step). The samples were then microwaved and stained with 95% ethanol containing eosin for 1 min 15 s, with the temperature kept around 60 °C. After that, dehydration was subsequently conducted with 100% ethanol at 60 °C, 1 min 15 s for two times in a microwave oven. Tissue transparency was achieved via infiltration with isopropanol at 58 °C for 1 min 30 s, then the samples were transferred to a solution of isopropanol and paraffin (v:v = 1:1) at 58 °C for 10 min. Isopropanol was then sequentially replaced by paraffin at 60 °C four times (30 min each time, with fresh solution used each time) also in a microwave oven. For details on the equipment assembly of the oven-heating water bath, please refer to Fig. S15D. Note: the microwave process required constant monitoring to avoid explosions through temperature measurement, especially during the paraffin steps. Once the temperature reached 5 °C above the expected temperature, the equipment needed to be taken out, and ice cubes had to be added to bring the water bath back to the desired temperature. Throughout all the above steps, the samples were fixed within a water bath controlled by a microwave oven. The microwave oven was necessary for the infiltration of different solutions. Finally, the samples were embedded in paraffin at room temperature.

After the samples were cooled, they were trimmed and sectioned. The thickness of all sample sections was 12 µm. After microscopic examination, the slides with target tissue were placed onto an RNase-free PEN membrane glass slide (Carl Zeiss, 415,190–9041 - 000). The sections were then spread at 42 °C, dried, and baked for 10 min at 42 °C before being stored at − 20 °C for long-term preservation. Laser capture microdissection (LCM) was performed using the ZEISS PALM MicroBeam system (Additional file 1: Fig. S15E). The samples were collected into a 600-μl enzyme-free Eppendorf tube (Axygen, MCT- 060-C-S). We recommend rapid collection to avoid RNA or DNA degradation. The tubes with dissected samples needed to be placed on ice and rapidly transferred into the library preparation steps. For each sample, two biological replicates were collected.

### RNA-seq library construction

The DNA of dissected tissues was isolated with RNeasy Plus Micro Kit (Qiagen, 74,034). Smart-seq v4 Ultra Low Input RNA Kit (Takara, 091817) was used for amplifying purified mRNA. Briefly, 9.5 μl input RNA mixed with 1 μl lysis buffer (0.95 μl 10 × Lysis buffer, 0.05 μl RNase Inhibitor) and 2 μl 3′SMART-Seq CDS Primer II A, mix well with pipetting, incubate the 200 µl PCR reaction tubes at 72 °C in a preheated thermocycler for 3 min followed by immediate cooling on ice for 2 min. For each tube, 5.5 μl Master Mix (4 μl 5 × Ultra Low First-Strand Buffer, 1 μl SMART-Seq v4 Oligonucleotide, 0.5 μl RNase Inhibitor) was added and mixed. Place the tubes in a thermocycler and run the following program: 42 °C 90 min, 70 °C 10 min, 4 °C. Prepare PCR Master Mix (25 μl 2 × SeqAmp PCR buffer, 1 μl PCR Primer II A, 1 μl SeqAmp DNA Polymerase, and 3 μl Nuclease-Free water for each reaction) and add it to each tube. Mix well and run the following programs: 95 °C 1 min, (98 °C 10 s, 65 °C 30 s, 68 °C 3 min) × 14 cycles, 72 °C 10 min, 4 °C. The amplified DNA was sheared to 200–500 bp range by sonication and purified with AMPure XP beads. One microgram DNA was used for following library construction. Purified DNA was end repaired, added with adenylate in the 3′ ends, ligated with adapters, and amplified with PCR for 7–8 cycles with the reagents from TruSeq® ChIP Sample Preparation Kit (Illumina). At last, DNA was purified with 0.8 × volume AMPure XP beads. The libraries were sequenced at Novogene Bioinformatics Institute on an Illumina HiSeq platform.

### RNA-seq data analysis

HISAT (v0.1.6) was used to map clean reads to the rice reference genome (RGAP version 7.0). Cufflinks (v2.2.1) and Cuffdiff (v2.2.1) were used to splice transcripts and find differentially expressed genes (DEGs). A strict criteria (fold change > 4, *q* value < 0.01) was used to filter DEGs.

Expression matrix for all samples (including data for root tip) [2, 27] was generated. Multidimensional scaling analysis (MDS) was performed to calculate the Euclidean distances of expression profiles of different samples, and scatter plot of distances was drawn in the R environment.

GO enrichment analysis was conducted by the software within the agriGO website (http://systemsbiology.cau.edu.cn/agriGOv2/).

Co-expression network modules were identified by the WGCNA package in the R environment. Genes with maximum RPKM > 2 and coefficient of variation > 1 were kept, and the retained 14,312 genes were used for the following analysis. Sixteen was calculated as a soft threshold using the “pickSoftThreshold” module of WGCNA, “blockwiseModules” module was used for the identification of co-expression network modules. In total, 29 modules were identified, and the heat maps of expression patterns of different modules were drawn by ggplot2 (v3.4.4) in the R environment.

Our data demonstrated good reproducibility between the two biological transcriptome replicates, and several genes essential for crown or lateral root development were identified through the RNA-seq data analysis and reverse genetic experiments. Therefore, we believe that the two biological replicates were sufficient for this study.

### BS-seq library construction

The DNA of dissected tissues was isolated with a DNA Micro Kit (Qiagen, 56,304). Bisulfite conversion was performed by utilizing the Zymo EZ Methylation Direct Kit (Zymo, D5020). Converted DNA was purified with PureLink PCR Purification Column Kit (Invitrogen, K210012) with minor modification (add M-Desulphonation Buffer to the column and hold for 15 min before washing) as Clark et al. (2017) suggested [45]. The converted ssDNA was then used for following BS-seq libraries construction with TruSeq® DNA Methylation Kit (Illumina). Briefly, mix 9 μl input DNA and 2 μl DNA Synthesis Primer thoroughly, heat to 95 °C in a thermocycler, and hold for 5 min followed by immediate cooling on ice. Add 5 μl Master Mix to each tube, and run the program in a thermocycler: 25 °C 5 min, 42 °C 30 min, 37 °C 2 min. Mix 1 μl exonuclease to each reaction, and run the program: 37 °C 10 min, 95 °C 3 min, 25 °C 2 min. Add 8 μl TT Master Mix to each tube and run the program: 25 °C 30 min, 95 °C 3 min, 4 °C hold. Purify DNA with AMPure XP beads and elute di-tagged DNA in 22.5 nuclease-free water. Prepare PCR reaction mixture (25 μl FailSafe PCR PreMix, 1 μl TruSeq DNA Methyl Forward, 1 μl Index PCR Primer, and 0.5 μl FailSafe PCR Enzyme Mix) and mix into each reaction and amplify DNA in a thermocycler as follows: 95 °C 1 min, (95 °C 30 s, 55 °C 30 s, 68 °C 3 min) × 16 cycles, 68 °C 7 min, 4 °C hold. Finally, DNA was purified with AMPure XP beads and sequenced by Novogene company.

### BS-seq data analysis

Clean reads were mapped to the rice reference genome (RGAP version 7.0) by BS-seeker2 (v2.1.8) [46]. Uniquely mapped reads excluding duplication were retained for further analysis. The coverage (Supplementary Table 2) of bisulfite-seq were calculated by (Number_unique_reads_of_replicate1_ + Number_unique_reads_of_replicate2_)* 150 bp* 2/373 million. Differentially methylation regions (DMRs) were calculated as follows: genome was binned into 50 bp windows, coverages (sum of sequenced cytosine depth) of each bin were counted, bins with at least 20 informative sequenced cytosines (the sum of two replicates) were retained for further analysis. The methylation level in each bin was determined by dividing the total number of methylated cytosines with sequenced cytosine numbers from the two replicates. Bins with methylation differences greater than 0.5, 0.3, and 0.1 at CG, CHG, and CHH sites, respectively, as well as *P* values (Fisher’s exact test) less than 0.05, were extracted. Considering replicate variance, only bins with methylation difference between any replicate comparison combination respectively larger than 0.5, 0.3, and 0.1 for CG, CHG, and CHH were finally considered as DMRs for further analysis.

For DMR-associated TE length statistics, 2000 randomly distributed 50 bp windows for each chromosome were selected (24,000 in total). The random window associated TE length was calculated as control.

### Assay of the association between transcription and DNA methylation

To identify the correlation between gene transcription and the corresponding promoter’s methylation, the CHH methylation of 200 bp bins within the 2 kb promoter regions was calculated. Pearson correlation coefficient between transcription and DNA methylation are summarized across all the tested samples. For each gene, the bin with the highest coefficient between its methylation and the corresponding gene transcription was defined as the most related methylated promoter region, and thereby this value was thought as the correlation coefficient between transcription and promoter methylation for the specific gene. A false discovery rate threshold of < 0.01 was used in this analysis.

CHH methylation of the promoter regions greater than 0.5 was defined as a modified promoter in Fig. S7.

### CUT&Tag coupled with qPCR

Briefly, the coleoptile nodes and roots containing initiating CR and LR primordia, were collected from the roots 4–5 and 7–10 days after germination, respectively. The nuclei were extracted by tissue cutting and meshing with the NEB2 buffer (250 mM sucrose, 10 mM Tris–Hcl pH 8.0, 1 mM Mgcl_2_, 0.1% TritonX- 100, 1 × protease inhibitor) with sequential filtering with 48 µm nylon membrane. 1/2 volume nuclei for each sample was take out for control assay (IgG). CUT&Tag procedure was strictly adhering the commercial kit protocol (TD903, Vazyme). Fifteen PCR cycles was utilized for micro DNA amplification. Mouse IgG (AC011, ABclonal) was applied for control samples, with flag antibody (M20008, Abmart) were used for experimental groups. DNA obtained from the CUT&Tag procedure was sequentially used as templates for the qPCR. These reactions were conducted by QuantStudio™ 3 System (Applied Biosystems) using Hieff UNICON® Universal Blue qPCR SYBR Green Master Mix (11184ES03, Yeasen) (Additional file 7: Table S6). The DNG702 binding strength to the target loci was calculated and normalized by anti-flag/IgG.

### Inspection of crown and lateral root phenotypes

The crown and lateral root numbers of 7-day-after-germination (DAG) wild type and knock-out line seedlings were counted. As for lateral root numbers, they were counted within the 1 cm region of the primary root starting from where the lateral root was just visible.

## Supplementary Information

Additional file 1. Fig. S1. Laser microdissection of initials of different root types. Fig. S2. Developmental stage alignments for ER, CR, and LR. Fig. S3. Expression patterns of reported marker genes involved in crown and lateral root development. Fig. S4. Identifying differentially expressed genes during root initiation. Fig. S5. Heat maps showing the expression patterns of each module clustered by WGCNA. Fig. S6. Global CG and CHG methylation are relatively stable during rice root initiation. Fig. S7. Characterization of differentially methylated regions. Fig. S8. CHH methylation dynamics of the transposon element within the promoter region correlated with gene transcription change. Fig. S9. Transcription profile of genes related to DNA methylation regulation. Fig. S10. Crown and/or lateral root initial numbers decreased in *ta2*, *OxDRM2*, and *bzip65*. Fig. S11. Analysis of misregulated genes in *ta2* mutant and *DRM2* overexpression lines. Fig. S12. DNA methylation affects functional genes in CR and LR. Fig. S13.*CBP* was identified as a potential functional gene for crown root development in rice. Fig. S14. Global correlation assay between CHH methylation and transcription.

Additional file 2. Table S1. RNA-seq analysis data.

Additional file 3. Table S2. BS-seq analysis data.

Additional file 4. Table S3. Differentially methylated regions identified in this study.

Additional file 5. Table S4. Gene list of which transcription positively or negatively correlated with promoter CHH methylation.

Additional file 6. Table S5. Gene information in this work.

Additional file 7. Table S6. Primers used in this study.

## Data Availability

The gene information mentioned in this study is listed in Supplementary Table 6. Gene information for these loci can be found on the Rice Genome Annotation Project website (http://rice.plantbiology.msu.edu/index.shtml). The gene sequences of CDS and protein, along with their corresponding annotations, are accessible via the ‘locus search’ function on the website. Data supporting the findings of this work (the RNA-seq and BS-seq data) have been deposited into the Gene Expression Omnibus database under the accession number GSE136767 [47] and GSE283466 [48]. The processed data from RNA-seq and BS-seq analyses are listed in Supplementary Table 1 and 2.
