# Supplementary material for: DNA methylation dynamics play crucial roles in shaping the distinct transcriptomic profiles for different root-type initiation in rice
Source: Genome Biol. 2025 Apr 17;26:99. doi: 10.1186/s13059-025-03571-0 (PMC12004658; doi:10.1186/s13059-025-03571-0)
Supplement: Supplementary file 2 — Additional file 2. Table S1. RNA-seq analysis data. [file 13059_2025_3571_MOESM2_ESM.docx]

| **Sample** | **Sample** | **replicate** | **Clean reads** | **pairs aligned exactly 1 time** | **Unique map ratio** | **Coefficient factor **** |
| --- | --- | --- | --- | --- | --- | --- |
| WT | CR-0 | 1 | 58,319,863 | 39,325,968 | 52.34% | 0.975 |
|  |  | 2 | 57,966,143 | 40,119,837 | 52.54% |  |
|  | CR-1 | 1 | 60,974,400 | 45,289,812 | 59.03% | 0.974 |
|  |  | 2 | 49,436,466 | 36,957,133 | 60.02% |  |
|  | CR-2 | 1 | 37,271,034 | 20,249,386 | 44.66% | 0.972 |
|  |  | 2 | 45,889,141 | 27,502,950 | 47.52% |  |
|  | CR-3 | 1 | 53,880,703 | 43,725,135 | 73.34% | 0.981 |
|  |  | 2 | 74,324,062 | 60,231,975 | 73.15% |  |
|  | LR-0 | 1 | 125,518,136 | 31,662,731 | 25.23% | 0.964 |
|  |  | 2 | 121,982,176 | 28,593,121 | 23.44% |  |
|  | LR-1 | 1 | 34,639,247 | 27,107,263 | 78.26% | 0.988 |
|  |  | 2 | 35,823,212 | 28,245,636 | 78.85% |  |
|  | LR-2 | 1 | 72,754,106 | 39,007,356 | 39.75% | 0.967 |
|  |  | 2 | 81,256,514 | 44,048,451 | 40.62% |  |
|  | LR-3 | 1 | 91,445,725 | 58,192,595 | 47.45% | 0.969 |
|  |  | 2 | 67,337,796 | 43,097,662 | 47.30% |  |
|  | ER-0 | 1 | 52,013,714 | 33,890,904 | 65.16% | 0.979 |
|  |  | 2 | 49,653,866 | 32,012,471 | 64.47% |  |
|  | ER-1 | 1 | 47,971,913 | 27,741,256 | 57.83% | 0.984 |
|  |  | 2 | 36,716,832 | 21,657,686 | 58.99% |  |
|  | ER-2 | 1 | 36,185,429 | 14,883,309 | 41.13% | 0.977 |
|  |  | 2 | 45,558,998 | 20,440,415 | 44.87% |  |
|  | ER-3 | 1 | 50,940,096 | 22,312,573 | 43.80% | 0.971 |
|  |  | 2 | 48,522,491 | 18,480,811 | 38.09% |  |
| *OxDRM2* | CR-1 | 1 | 58,562,304 | 50,370,903 | 85.70% | 0.999 |
|  |  | 2 | 56,105,371 | 48,586,647 | 86.48% |  |
|  | LR-1 | 1 | 58,282,755 | 40,243,991 | 76.64% | 0.998 |
|  |  | 2 | 58,483,970 | 42,726,417 | 72.58% |  |
| *dng702/ta2* | CR-1 | 1 | 52,350,505 | 39.536.399 | 75.33% | 0.999 |
|  |  | 2 | 64,565,738 | 50,224,703 | 75.91% |  |
|  | LR-1 | 1 | 51,534,748 | 28,810,206 | 61.35% | 0.999 |
|  |  | 2 | 52,125,417 | 34,897,377 | 62.55% |  |

**Supplementary Table 1. RNA-seq analysis data**
