# Supplementary material for: DNA methylation dynamics play crucial roles in shaping the distinct transcriptomic profiles for different root-type initiation in rice
Source: Genome Biol. 2025 Apr 17;26:99. doi: 10.1186/s13059-025-03571-0 (PMC12004658; doi:10.1186/s13059-025-03571-0)
Supplement: Supplementary file 3 — Additional file 3. Table S2. BS-seq analysis data. [file 13059_2025_3571_MOESM3_ESM.docx]

**Supplementary Table 2. BS-seq analysis data**

|  | **Sample** | **Raw reads** | **Unique mapped reads** | **Unique mapped ratio** | **Coverage** | **Coefficient factor** |
| --- | --- | --- | --- | --- | --- | --- |
| WT | CR-0-1 | 33,449,144 | 20,475,836 | 61.2148% | 35.42× | 0.985 |
|  | CR-0-2 | 38,469,587 | 23,597,024 | 61.3394% |  |  |
|  | CR-1-1 | 39,324,473 | 23,042,742 | 58.5964% | 34.76× | 0.984 |
|  | CR-1-2 | 34,469,387 | 20,208,842 | 58.6284% |  |  |
|  | CR-2-1 | 31,710,319 | 18,308,486 | 57.7367% | 28.83× | 0.975 |
|  | CR-2-2 | 30,313,918 | 17,559,890 | 57.9268% |  |  |
|  | LR-0-1 | 53,253,329 | 32,211,888 | 60.4880% | 46.93× | 0.930 |
|  | LR-0-2 | 43,150,224 | 26,180,113 | 60.6720% |  |  |
|  | LR-1-1 | 56,872,950 | 29,623,595 | 52.0873% | 43.45× | 0.915 |
|  | LR-1-2 | 46,424,189 | 24,428,683 | 52.6206% |  |  |
|  | LR-2-1 | 45,107,844 | 26,376,083 | 58.4734% | 44.02× | 0.968 |
|  | LR-2-2 | 48,268,807 | 28,394,850 | 58.8265% |  |  |
|  | ER-0-1 | 45,491,801 | 28,053,751 | 61.6677% | 50.06× | 0.977 |
|  | ER-0-2 | 56,670,789 | 34,231,939 | 60.4049% |  |  |
|  | ER-1-1 | 49,652,439 | 29,915,803 | 60.2504% | 48.71× | 0.986 |
|  | ER-1-2 | 49,083,553 | 30,688,585 | 62.5232% |  |  |
|  | ER-2-1 | 40,462,949 | 24,545,137 | 60.6608% | 41.65× | 0.985 |
|  | ER-2-2 | 44,687,401 | 27,268,342 | 61.0202% |  |  |
|  | **Sample** | **Raw reads** | **Unique mapped reads** | **Unique mapped ratio** | **Coverage** | **Coefficient factor** |
| *OxDRM2* | CR-1-1 | 43,727,622 | 26,009,930 | 29.6217% | 43.33× | 0.985 |
|  | CR-1-2 | 46,750,856 | 27,893,847 | 28.9963% |  |  |
|  | LR-1-1 | 40,980,160 | 23,947,492 | 26.2597% | 27.27× | 0.972 |
|  | LR-1-2 | 36,941,442 | 9,990,816 | 27.0540% |  |  |
| *dng702/ta2* | CR-1-1 | 59,278,253 | 16,594,698 | 28.0044% | 26.74× | 0.978 |
|  | CR-1-2 | 59,914,405 | 16,668,746 | 27.8209% |  |  |
|  | LR-1-1 | 59,515,815 | 15,722,507 | 26.4174% | 25.16× | 0.973 |
|  | LR-1-2 | 56,536,210 | 15,582,797 | 27.5625% |  |  |
